# Feasibility of Telemonitoring Blood Pressure in Patients With Kidney Disease (Oxford Heart and Renal Protection Study-1): Observational Study

**DOI:** 10.2196/11332

**Published:** 2018-12-21

**Authors:** Bronwen E Warner, Carmelo Velardo, Dario Salvi, Kathryn Lafferty, Sarah Crosbie, William G Herrington, Richard Haynes

**Affiliations:** 1 Oxford University Hospitals National Health Service Foundation Trust Oxford United Kingdom; 2 Institute of Biomedical Engineering University of Oxford Oxford United Kingdom; 3 Medical Research Council Population Health Research Unit Nuffield Department of Population Health Oxford United Kingdom; 4 Clinical Trial Service Unit and Epidemiological Studies Unit Nuffield Department of Population Health Oxford United Kingdom

**Keywords:** chronic kidney disease, blood pressure, telemonitoring, mobile phone

## Abstract

**Background:**

Blood pressure (BP) is a key modifiable risk factor for patients with chronic kidney disease (CKD), with current guidelines recommending strict control to reduce the risk of progression of both CKD and cardiovascular disease. Trials involving BP lowering require multiple visits to achieve target BP, which increases the costs of such trials, and in routine care, BP measured in the clinic may not accurately reflect the usual BP.

**Objective:**

We sought to assess whether a telemonitoring system for BP (using a Bluetooth-enabled BP machine that could transmit BP measurements to a tablet device installed with a bespoke app to guide the measurement of BP and collect questionnaire data) was acceptable to patients with CKD and whether patients would provide sufficient BP readings to assess variability and guide treatment.

**Methods:**

A total of 25 participants with CKD were trained to use the telemonitoring equipment and asked to record BP daily for 30 days, attend a study visit, and then record BP on alternate days for the next 60 days. They were also offered a wrist-worn applanation tonometry device (BPro) which measures BP every 15 minutes over a 24-hour period. Participants were given questionnaires at the 1- and 3-month time points; the questionnaires were derived from the System Usability Scale and Technology Acceptance Model. All eligible participants completed the study.

**Results:**

Mean participant age was 58 (SD 11) years, and mean estimated glomerular filtration rate was 36 (SD 13) mL/min/1.73m2. 13/25 (52%) participants provided >90% of the expected data and 18/25 (72%) provided >80% of the expected data. The usability of the telemonitoring system was rated highly, with mean scores of 84.9/100 (SE 2.8) after 30 days and 84.2/100 (SE 4.1) after 90 days. The coefficient of variation for the variability of systolic BP telemonitoring was 9.4% (95% CI 7.8-10.9) compared with 7.9% (95% CI 6.4-9.5) for the BPro device, *P*=.05 (and was 9.0% over 1 year in a recently completed trial with identical eligibility criteria), indicating that most variation in BP was short term.

**Conclusions:**

Telemonitoring is acceptable for patients with CKD and provides sufficient data to inform titration of antihypertensive therapies in either a randomized trial setting (comparing BP among different targets) or routine clinical practice. Such methods could be employed in both scenarios and reduce costs currently associated with such activities.

**Trial Registration:**

International Standard Randomized Controlled Trial Number ISRCTN13725286; http://www.isrctn.com/ISRCTN13725286 (Archived by WebCite at http://www.webcitation.org/74PAX51Ji).

## Introduction

Chronic kidney disease (CKD) is estimated to affect between 5% and 14% of the adult population worldwide [1,2] and is strongly associated with cardiovascular disease (CVD) [3,4]. Blood pressure (BP) rises in patients with CKD due to salt and water retention, increased sympathetic nervous system activity, activation of the renin-angiotensin-aldosterone system, and reduction in endogenous vasodilators. BP has a strong positive association with cardiovascular events in the general population [5] and among patients with CKD (once confounding by prior CVD is properly accounted for) [6]. Therefore, the relationship between raised BP and CKD and the potential for therapeutic interventions addressing BP in this population are an important focus of research.

The beneficial impact of BP lowering in the CKD population has not yet been fully established. First, it has been suggested that BP lowering reduces the risk of progression among patients with proteinuric CKD [7]. However, it is uncertain whether BP is causally related to the progression of CKD, and overall, it is not clear whether BP lowering reduces the risk of progression [8]. Second, the benefit of BP lowering on CVD in patients with CKD has not yet been fully elucidated. In the general population, lowering systolic BP (SBP) by 10 mm Hg reduces the risk of cardiovascular events by about 20% [8]. There is some evidence that this treatment effect is attenuated among patients with CKD (relative risk per 10 mm Hg reduction among patients with CKD 0.84, CI 0.73-0.96; relative risk per 10 mm Hg reduction among patients without CKD 0.68, CI 0.62-0.75; *P* value for interaction=.01); however, few patients with more advanced CKD (eg, estimated glomerular filtration rate [eGFR]<45 mL/min/1.73 m^2^) were included in these trials [8,9]. Finally, the safety of BP lowering in patients with CKD is less well established in part due to the higher baseline risk of acute kidney injury (a recognized hazard of intensive BP lowering) among patients with CKD [10]. Studies are needed to assess BP lowering in patients with advanced CKD and to compare more- versus less-intensive BP reduction, but such trials are potentially expensive in part due to the requirement for frequent visits to measure BP and titrate BP-lowering treatment.

Nevertheless, BP control remains a key focus of those managing patients with CKD. BP measurements in the clinical setting are somewhat imprecise measures of long-term average BP (because of “white-coat” and “masked” hypertension [11,12]), and clinic measurements do not detect short- to medium-term within- person BP variability. A method through which BP can be measured frequently at home may, therefore, be of utility to clinical teams and those designing and conducting trials.

Home BP monitoring, as an intervention, has previously been the subject of trials looking to improve BP control [13,14]. It has been shown that self-monitoring improves BP control [15]. Telemonitoring is an evolving topic of interest in the management of chronic conditions including hypertension [16], with evidence of acceptability in certain patient groups [17]. Telemonitoring technology is now available, which allows such home measurements to be automatically transferred to a central computer where they can be reviewed by and responded to by medical staff as necessary. Telemonitoring has now been proposed as a novel approach for data collection in trials involving BP lowering for patients with CKD and as an enhancement to standard clinical care. This feasibility study aims to examine the potential for BP telemonitoring in terms of participant acceptability and consequent concordance with the technology infrastructure for patients with advanced CKD with the long-term goal of randomized trials investigating BP lowering in this cohort and improving routine clinical care.

## Methods

### Aims

The primary aim of the study was to assess the participants’ acceptability of BP telemonitoring over 3 months, determined by the proportion of patients providing at least 90% of expected data. Expected data were defined as daily readings for 30 days and alternate daily readings for a further 60 days.

The 4 secondary aims were as follows: (1) to examine the usability and tolerability of the telemonitoring system as assessed using a questionnaire at the 1- and 3-month time points; (2) to determine the intraindividual variability in BP; (3) to quantify the proportion of patients reaching target BP by follow-up; and (4) to compare the telemonitoring measurements (and their variability) with those taken by an applanation tonometry device (“BPro”) [18,19] that measures BP every 15 minutes over 24 hours in a subset of participants. Patients were eligible if they had evidence of CKD at risk of progression (detailed eligibility criteria and participant flow diagram can be found in Multimedia Appendices 1 and 2) [20].

### Study Methodology

The study consisted of 2 phases: intensive monitoring and titration. During the first month intensive monitoring phase, participants were asked to measure their BP daily after resting for 5 minutes, with 3 measurements on each occasion. Changes in BP medication were avoided during this period, unless required by a local clinical team. At the end of the intensive monitoring phase, participants were offered a BPro device to wear for 24 hours. Irrespective of the use of the BPro device, a titration phase followed lasting a further 2 months. During titration, participants were instructed to reduce the frequency of their BP measurements to alternate days, and additional antihypertensive agents were introduced, as necessary, by a study clinician, according to concomitant medications and comorbidities, with a target SBP of <140 mm Hg (urine albumin/creatinine ratio <3 mg/mmol) or <130 mm Hg (urine albumin/creatinine ratio ≥3 mg/mmol); ie, according to current clinical guidelines [21]. At the 1-month and 3-month time points, participants attended the Unit and were asked to complete a questionnaire assessing their confidence in using the telemonitoring system and its acceptability. The questionnaire included 2 sections, one derived from the System Usability Scale [22], asked at 1 and 3 months, and a second derived from the Technology Acceptance Model [23], asked only at month 3 (Multimedia Appendix 3). Statistical methods can be found in Multimedia Appendix 1.

### Telemonitoring System

Participants were provided with an “off-the-shelf”, Bluetooth- enabled BP monitor (A&D medical UA-767PBT-Ci) and a tablet computer with custom-developed software (“app”; Multimedia Appendix 4). Patients used the app to receive instructions about the measurements and then used the monitor to measure their BP. Readings were transferred wirelessly from the BP monitor to the tablet computer. Shortly after, the tablet computer would synchronize with the study central system. The central system was hosted by the Oxford University Hospitals National Health Service Foundation Trust and was managed by the local Information Management and Technology team. Its software comprised a database, where all the data were stored, and a password-protected Web interface for the study management. The interface allowed researchers to remotely monitor the home-recorded BP readings and the completed symptom questionnaires. Mobile internet connection was required for the readings to be transferred. The mobile app was capable of storing data in case of no connectivity and up until the tablet computer was in a location with sufficient internet access. If participants had a problem recording their measurements, they were able to contact the coordinating center during working hours. If the coordinating center did not receive BP readings within 5 days, the participant was contacted to identify and seek to resolve any problems.

## Results

Between June 2016 and April 2017, 25 patients were recruited. Mean participant age was 58 (SD 11.0) years, with about half the cohort being >60 years old (Table 1). Among the 25 participants, 21 (84%) were male. The average BP at entry was 152/82 mm Hg and the mean eGFR was 36 (SD 13.3) mL/min/1.73 m^2^. The most common primary causes of CKD were diabetes, glomerulonephritis, and hypertension.

**Figure 1:**
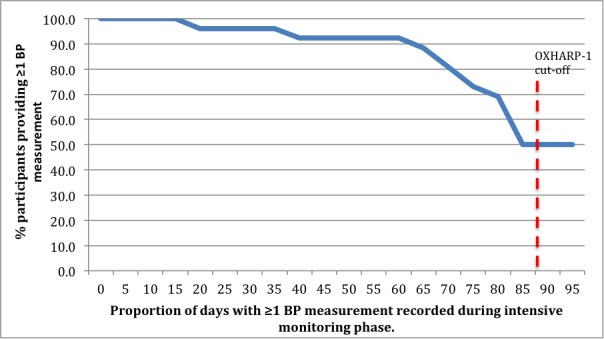
Primary aim: proportion of days with at least 1 blood pressure (BP) measurement during intensive monitoring phase. OXHARP-1: Oxford Heart and Renal Protection Study-1.

Among the 25 participants, 18 (72%) provided >80% of the expected data and 13 (52%) provided >90% of the expected data throughout the whole study period (Figure 1). The results were similar for the intensive monitoring and titration phases, with 52% (13/25) subjects providing >90% expected data at both time points. BP data provided according to baseline characteristics are shown in Multimedia Appendix 5. The average number of readings provided via BPro (among the 13 participants who accepted it) was 51 (out of a maximum of 96; ranging from 7 to 75).

The telemonitoring system was found to be a generally acceptable method to record home BP, with mean (SE) System Usability Scale score of 84.9 (2.8) after the 1-month intensive monitoring phase (Multimedia Appendix 6). After the 2-month titration phase, the mean score was 84.2 (4.1). At the end of the study, an additional 6 questions assessing overall participant impressions of telemonitoring were asked (Multimedia Appendix 7), demonstrating good overall acceptance.

**Figure 2:**
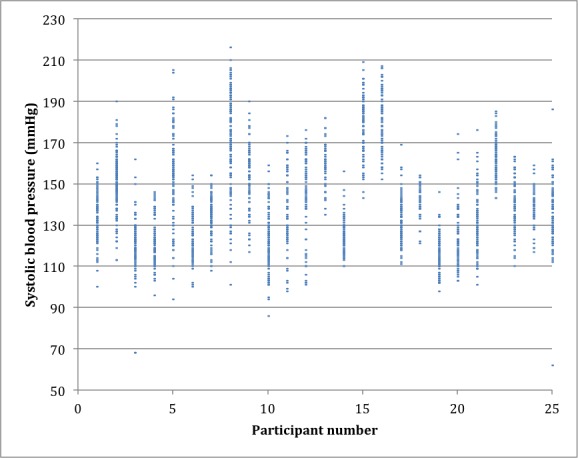
Individual systolic blood pressure measurements over the 3-month study period.

Intraindividual variability was calculated for each participant over the intensive monitoring phase. Among all participants, means of the SD values of intraindividual SBP and diastolic BP (DBP) were 13.8 and 7.4 mm Hg, respectively (Figure 2 and Multimedia Appendix 8). Among the 13 participants who accepted a BPro device, the mean BPro intraindividual SD (over 24 hours) was 10.4 mm Hg for SBP and 6.1 mm Hg for DBP. The SBP coefficient of variation for telemonitoring versus BPro was 9.4% (95% CI 7.8-10.9) versus 7.9% (95% CI 6.4-9.5; *P*=.05), and the DBP coefficient of variation for telemonitoring versus BPro was 9.7% (95% CI 7.2-11.5) versus 7.4% (95% CI 6.5-8.3; *P*=.01; Multimedia Appendix 9). The average SBP values provided by BPro and the telemonitoring systems were similar: telemonitoring mean SBP 140.6 mm Hg versus BPro mean SBP 138.1 mm Hg; telemonitoring mean DBP 80.1 mm Hg versus BPro mean DBP 83.6 mm Hg. At the individual participant level, the mean SBP difference was +3.1 mm Hg (SE 4.7) and the mean DBP difference was −3.4 mm Hg (SE 2.1).

A *post hoc* analysis showed that the coefficient of variation for SBP measured using telemonitoring over 1 week was 8.3% (95% CI 5.6-10.9), similar to that for a longer period.

There was an improvement in the proportion of patients in target BP range over the course of the study from 3 to 9. The mean SBP at baseline, 152.5 (SD 16.2) mm Hg, reduced to 138.52 (SD 14.3) mm Hg at 3 months (mean individual change −14.0 [SE 3.7] mm Hg). A change in dose (5 participants) or choice (8 participants) of antihypertensive medication was made in 13 of 25 subjects.

**Table 1:** Baseline characteristics of the participants.

Baseline characteristics		Participants
**Age in years, mean (SD)**		58 (11)
	<40, n (%)	2 (8)
	≥40 to <60, n (%)	11 (44)
	≥60, n (%)	12 (48)
**Sex, n (%)**		
	Male	21 (84)
	Female	4 (16)
**Systolic blood pressure in mm Hg, mean (SD)**		152 (16)
	<130, n (%)	2 (8)
	≥130 to <150, n (%)	6 (24)
	≥150, n (%)	17 (68)
**Diastolic blood pressure in mm Hg, mean (SD)**		82 (13)
	<80, n (%)	11 (44)
	≥80 to <90, n (%)	7 (28)
	≥90, n (%)	7 (28)
**Estimated glomerular filtration rate in mL/min/1.73 m^**2**^, mean (SD)**		36 (13)
	<30, n (%)	11 (44)
	≥30 to <45, n (%)	9 (36)
	≥45, n (%)	5 (20)
**Urine albumin:creatinine ratio in mg/mmol, median (interquartile range)**		36.6 (101)
	<3, n (%)	9 (36)
	≥3 to <30, n (%)	1 (4)
	≥30, n (%)	15 (60)
**Cause of kidney disease, n (%)**		
	Diabetes	5 (20)
	Glomerulonephritis	5 (20)
	Hypertension	3 (12)
	Other or unknown	12 (48)
**Smartphone owner, n (%)**		
	Yes	17 (68)
	No	8 (32)

## Discussion

The Oxford Heart and Renal Protection Study-1 suggests that telemonitoring among patients with CKD is feasible and well tolerated. Telemonitoring has previously been used in the management of heart failure [24], type 2 diabetes [25], and hypertension [13]. This is the first feasibility study looking at BP telemonitoring as a potential data collection method for randomized controlled trials in patients with CKD.

Recording of regular BP readings is beneficial in both routine care and clinical trials. Home monitoring reduces the need for clinic visits, which can be time consuming and expensive for both the participant and clinical center, with suggestions that telehealth interventions such as telemonitoring have an overall favorable cost-effectiveness profile [26]. Furthermore, central analysis of BP readings can improve safety by allowing clinicians to instigate more timely interventions in cases of prolonged hypo- or hypertension. To assess the primary aim of participants’ acceptability of BP telemonitoring over 3 months, a threshold was set at subjects providing >90% of the expected BP data (54 readings in a 90-day period). Accordingly, 52% (13/25) of the subjects provided the target of >90% possible data, but when the threshold was set at 80%, 72% (18/25) of the subjects provided the requisite data and 92% (23/25) of subjects provided >65% of the possible BP readings. Therefore, this system provides sufficiently complete data to support either randomized trials or clinical care.

Our quantitative data are supported by questionnaire data, which suggests that participants found the technology usable. Estimation of longer-term acceptance also scored high, with all analyzed constructs being evaluated with acceptable results. Patients who owned a smartphone, that is, who are likely to be more confident with similar technology, were more positive in almost every area, while patients without a smartphone increased their questionnaire score from the 1-month to the 3-month time point. This may suggest that additional education of patients without smartphones before using similar equipment could be beneficial.

Telemonitoring equips patients with knowledge, skills, and technology to facilitate shared responsibility for their health care management. This must be balanced against the potential reduced patient-clinician contact and consequent missed opportunities to identify concerns. In order for a satisfactory quantity of data to be obtained so that home BP monitoring is feasible, participants must be able to understand and feel comfortable with the technology.

Recording and analysis of large numbers of BP readings by the patients at home allows for assessment of BP variability and gives a more accurate indication of a patient’s true usual (ie, long-term average) BP status rather than isolated clinic readings [27]. As expected, there was considerable variability in the BP values provided by each individual over the period of monitoring. In order to compare the telemonitoring system with other available BP measurement technologies, the BPro system was given to a sample of our patients. The 2 systems produced similar overall average SBP and DBP measurements, although telemonitoring samples BP over a 1-month period compared with just 24 hours for BPro. The variability observed with telemonitoring was also similar to that observed over a 1-year period in the UK Heart and Renal Protection-III trial [28], which had identical inclusion criteria and found coefficients of variation of 9.0% and 9.0% for SBP and DBP, respectively. These results suggest that variability in BP is largely short term, with little additional variability over longer periods, suggesting that variability can be measured over relatively short periods [6].

The BP variability seen in our cohort indicates that more intensive monitoring would be of use in both trial settings and routine clinical care. Furthermore, BP variability may be associated with cardiovascular and mortality outcomes over and above the effect of mean BP [29,30], and thus, measuring this variability may refine risk prediction models.

The number of participants in our study was small, in keeping with a pilot study, which may limit generalizability of the results. Follow-up was only for 3 months; thus, we did not assess how durable the compliance with the study protocol would be in a long-term trial or routine clinical care. Telemonitoring in combination with clinical review of BP measurements and consequent modification of antihypertensive medication has previously been shown to have efficacy in improving BP control [14,30]. However, not all trials involving telemonitoring have achieved overall reduction in BP, and a longer trial duration is needed to assess whether this positive outcome is sustainable. Future work could aim to investigate the effect of an intensive versus standard BP-lowering strategy (using telemonitoring to monitor and direct treatment) on kidney function.

We found that the telemonitoring technology is a practical means of collecting large amounts of BP data with the additional benefit of enabling the recording of BP variability. BP telemonitoring, therefore, has potential in both future studies of CVD management and in improvement of routine clinical care in this at-risk group.

## Appendix

Multimedia Appendix 1 Supplementary methods: eligibility criteria and statistical methods.

Multimedia Appendix 2 Participant flow.

Multimedia Appendix 3 Participant questionnaires.

Multimedia Appendix 4 Screenshot of telemonitoring intervention as viewed by participants, illustrating trend of blood pressure and heart rate over two-week period.

Multimedia Appendix 5 Blood pressure data provided according to baseline demographics.

Multimedia Appendix 6 System usability scale (SUS) scores at one month and three months.

Multimedia Appendix 7 Three-month questionnaire scores assessing overall participant impressions of telemonitoring at the end of the trial.

Multimedia Appendix 8 Individual diastolic blood pressure over the three-month trial period.

Multimedia Appendix 9 Individual systolic blood pressure according to the BPro device (over 24 hours) and the telemonitoring system (over one week at the period of the BPro measurements) for 13 patients.

## Abbreviations

BP: blood pressure
CKD: chronic kidney disease
CVD: cardiovascular disease
DBP: diastolic blood pressure
eGFR: estimated glomerular filtration rate
SBP: systolic blood pressure

## References

[1] Public Health England (2014). Chronic kidney disease prevalence model.

[2] Centers for Disease Control and Prevention National Health and Nutrition Examination Survey.

[3] Go AS, Chertow GM, Fan D, McCulloch CE, Hsu C (2004). Chronic kidney disease and the risks of death, cardiovascular events, and hospitalization. N Engl J Med.

[4] Matsushita K, van der Velde M, Astor BC, Woodward M, Levey AS, de Jong PE, Coresh J, Gansevoort RT, Chronic Kidney Disease Prognosis Consortium (2010). Association of estimated glomerular filtration rate and albuminuria with all-cause and cardiovascular mortality in general population cohorts: a collaborative meta-analysis. Lancet.

[5] Lewington S, Clarke R, Qizilbash N, Peto R, Collins R, Prospective Studies Collaboration (2002). Age-specific relevance of usual blood pressure to vascular mortality: a meta-analysis of individual data for one million adults in 61 prospective studies. Lancet.

[6] Herrington W, Staplin N, Judge PK, Mafham M, Emberson J, Haynes R, Wheeler DC, Walker R, Tomson C, Agodoa L, Wiecek A, Lewington S, Reith CA, Landray MJ, Baigent C, SHARP Collaborative Group (2017). Evidence for Reverse Causality in the Association Between Blood Pressure and Cardiovascular Risk in Patients With Chronic Kidney Disease. Hypertension.

[7] Lv J, Ehteshami P, Sarnak MJ, Tighiouart H, Jun M, Ninomiya T, Foote C, Rodgers A, Zhang H, Wang H, Strippoli GFM, Perkovic V (2013). Effects of intensive blood pressure lowering on the progression of chronic kidney disease: a systematic review and meta-analysis. CMAJ.

[8] Ettehad D, Emdin CA, Kiran A, Anderson SG, Callender T, Emberson J, Chalmers J, Rodgers A, Rahimi K (2016). Blood pressure lowering for prevention of cardiovascular disease and death: a systematic review and meta-analysis. Lancet.

[9] Ninomiya T, Perkovic V, Turnbull F, Neal B, Barzi F, Cass A, Baigent C, Chalmers J, Li N, Woodward M, MacMahon S, Blood Pressure Lowering Treatment Trialists' Collaboration (2013). Blood pressure lowering and major cardiovascular events in people with and without chronic kidney disease: meta-analysis of randomised controlled trials. BMJ.

[10] Wright JT, Williamson JD, Whelton PK, Snyder JK, Sink KM, Rocco MV, Reboussin DM, Rahman m, Oparil S, Lewis CE, Kimmel PL, Johnson KC, Goff DC, Fine LJ, Cutler JA, Cushman WC, Cheung AK, Ambrosius WT, SPRINT Research Group (2015). A Randomized Trial of Intensive versus Standard Blood-Pressure Control. N Engl J Med.

[11] Pickering TG, Gerin W, Schwartz AR (2002). What is the white-coat effect and how should it be measured?. Blood Press Monit.

[12] Pickering TG, Eguchi K, Kario K (2007). Masked hypertension: a review. Hypertens Res.

[13] Zullig LL, Melnyk SD, Goldstein K, Shaw RJ, Bosworth HB (2013). The role of home blood pressure telemonitoring in managing hypertensive populations. Curr Hypertens Rep.

[14] Orozco-Beltran D, Sánchez-Molla M, Sanchez JJ, Mira JJ, ValCrònic Research Group (2017). Telemedicine in Primary Care for Patients With Chronic Conditions: The ValCrònic Quasi-Experimental Study. J Med Internet Res.

[15] McManus RJ, Mant J, Franssen M, Nickless A, Schwartz C, Hodgkinson J, Bradburn P, Farmer A, Grant S, Greenfield SM, Heneghan C, Jowett S, Martin U, Milner S, Monahan M, Mort S, Ogburn E, Perera-Salazar R, Shah SA, Yu L, Tarassenko L, Hobbs FDR, TASMINH4 investigators (2018). Efficacy of self-monitored blood pressure, with or without telemonitoring, for titration of antihypertensive medication (TASMINH4): an unmasked randomised controlled trial. Lancet.

[16] Paré G, Moqadem K, Pineau G, St-Hilaire C (2010). Clinical effects of home telemonitoring in the context of diabetes, asthma, heart failure and hypertension: a systematic review. J Med Internet Res.

[17] Albrecht L, Wood P, Fradette M, McAlister F, Rabi D, Boulanger P, Padwal R (2018). Usability and Acceptability of a Home Blood Pressure Telemonitoring Device Among Community-Dwelling Senior Citizens With Hypertension: Qualitative Study. JMIR Aging.

[18] Williams B, Lacy PS, Yan P, Hwee C, Liang C, Ting C (2011). Development and validation of a novel method to derive central aortic systolic pressure from the radial pressure waveform using an n-point moving average method. J Am Coll Cardiol.

[19] (2017). HealthStats.

[20] Rothwell P, Howard S, Dolan E, O'Brien E, Dobson J, Dahlöf B, Sever P, Poulter N (2010). Prognostic significance of visit-to-visit variability, maximum systolic blood pressure, and episodic hypertension. Lancet.

[21] KDIGO (2012). KDIGO Clinical Practice Guideline for the Management of Blood Pressure in Chronic Kidney Disease. Kidney International Supplements.

[22] Brooke J (1996). SUS-A quick and dirty usability scale. Usability evaluation in industry.

[23] Gagnon MP, Orruño E, Asua J, Abdeljelil AB, Emparanza J (2012). Using a modified technology acceptance model to evaluate healthcare professionals' adoption of a new telemonitoring system. Telemed J E Health.

[24] Yun JE, Park J, Park H, Lee H, Park D (2018). Comparative Effectiveness of Telemonitoring Versus Usual Care for Heart Failure: A Systematic Review and Meta-analysis. J Card Fail.

[25] Iljaž Rade, Brodnik A, Zrimec T, Cukjati I (2017). E-healthcare for Diabetes Mellitus Type 2 Patients - A Randomised Controlled Trial in Slovenia. Zdr Varst.

[26] Chen Y, Lin Y, Hung C, Huang C, Yeih D, Chuang P, Ho Y, Chen M (2013). Clinical outcome and cost-effectiveness of a synchronous telehealth service for seniors and nonseniors with cardiovascular diseases: quasi-experimental study. J Med Internet Res.

[27] National Institute for Health and Care Excellence (2016). Hypertension in adults: diagnosis and management.

[28] UK HARP-III Collaborative Group (2017). Randomized multicentre pilot study of sacubitril/valsartan versus irbesartan in patients with chronic kidney disease: United Kingdom Heart and Renal Protection (HARP)- III-rationale, trial design and baseline data. Nephrol Dial Transplant.

[29] Stevens S, Wood S, Koshiaris C, Law K, Glasziou P, Stevens R, McManus RJ (2016). Blood pressure variability and cardiovascular disease: systematic review and meta-analysis. BMJ.

[30] Kim M, Han H, Hedlin H, Kim J, Song H, Kim K, Hill M (2011). Teletransmitted monitoring of blood pressure and bilingual nurse counseling-sustained improvements in blood pressure control during 12 months in hypertensive Korean Americans. J Clin Hypertens (Greenwich).

