# Supplementary material for: Feasibility of Telemonitoring Blood Pressure in Patients With Kidney Disease (Oxford Heart and Renal Protection Study-1): Observational Study
Source: JMIR Cardio. 2018 Dec 21;2(2):e11332. doi: 10.2196/11332 (PMC6309686; doi:10.2196/11332)
Supplement: Multimedia Appendix 1 [file cardio_v2i2e11332_app1.pdf]

SUPPLEMENTARY METHODS

Eligibility criteria

Inclusion criteria were all three of the following:

1. Patients with established CKD:
   - eGFR ≥20 <45 mL/min/1.73m^2; or
   - eGFR ≥45 <60 mL/min/1.73m^2 and urine albumin:creatinine ratio >20 mg/mmol or protein: creatinine ratio >30 mg/mmol;
2. Age >18 years;
3. Mean systolic BP at screening >130 mmHg

Exclusion criteria were any of the following: a planned change to BP lowering therapy within the next month; an acute vascular event (e.g. acute coronary syndrome, transient ischaemic attack or stroke) within the last month; or a medical history that might limit the participant's ability to comply with study procedures for the duration of the study.

Potentially eligible patients were identified from the local hospital database and invited to attend a screening clinic appointment at the Oxford Kidney Unit, in which medical history, relevant current treatment and eligibility factors were recorded. Written informed consent was sought from eligible and willing participants and telemonitoring equipment was provided along with training in its use. A sample of 25 participants was considered sufficient to test feasibility in a range of people.

Statistical methods

Because variability in BP is correlated with mean BP, BP variability was assessed by calculating the coefficient of variation (CV), where CV = 100 x SD/mean. Although this is not completely independent of the mean itself it was used as more advanced measures require modelling which were considered potentially unreliable due to the

small size of this cohort. Comparison of CVs between telemonitoring and BPro measurements was done by a paired t test.
