# Supplementary figures and images for: Feasibility of Telemonitoring Blood Pressure in Patients With Kidney Disease (Oxford Heart and Renal Protection Study-1): Observational Study
Source: JMIR Cardio. 2018 Dec 21;2(2):e11332. doi: 10.2196/11332 (PMC6309686; doi:10.2196/11332)

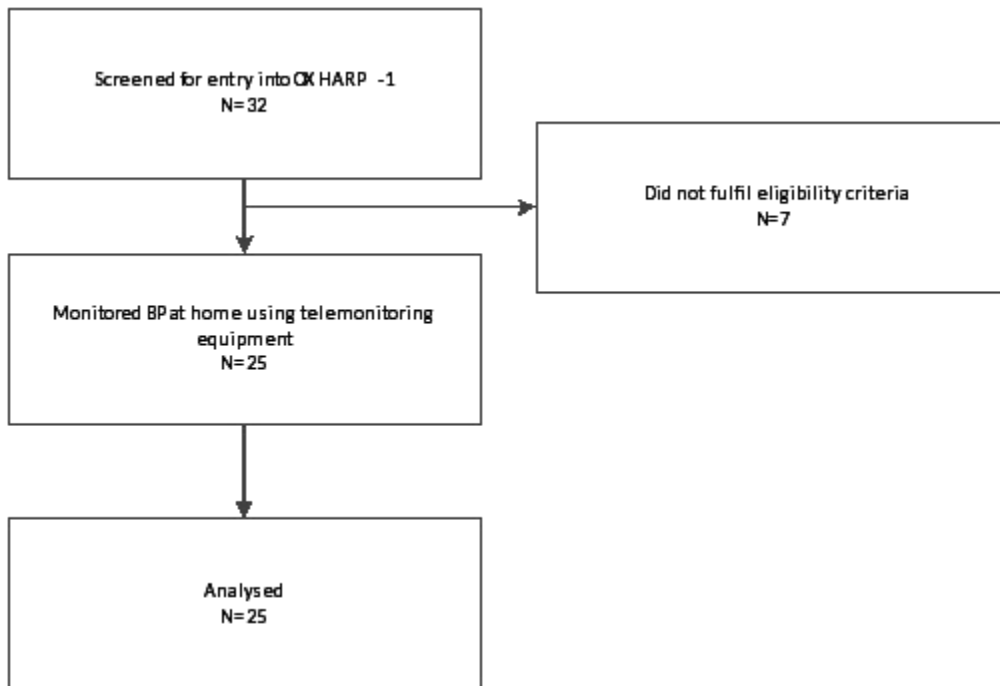

Supplement: Multimedia Appendix 2 [file cardio_v2i2e11332_app2.pdf]

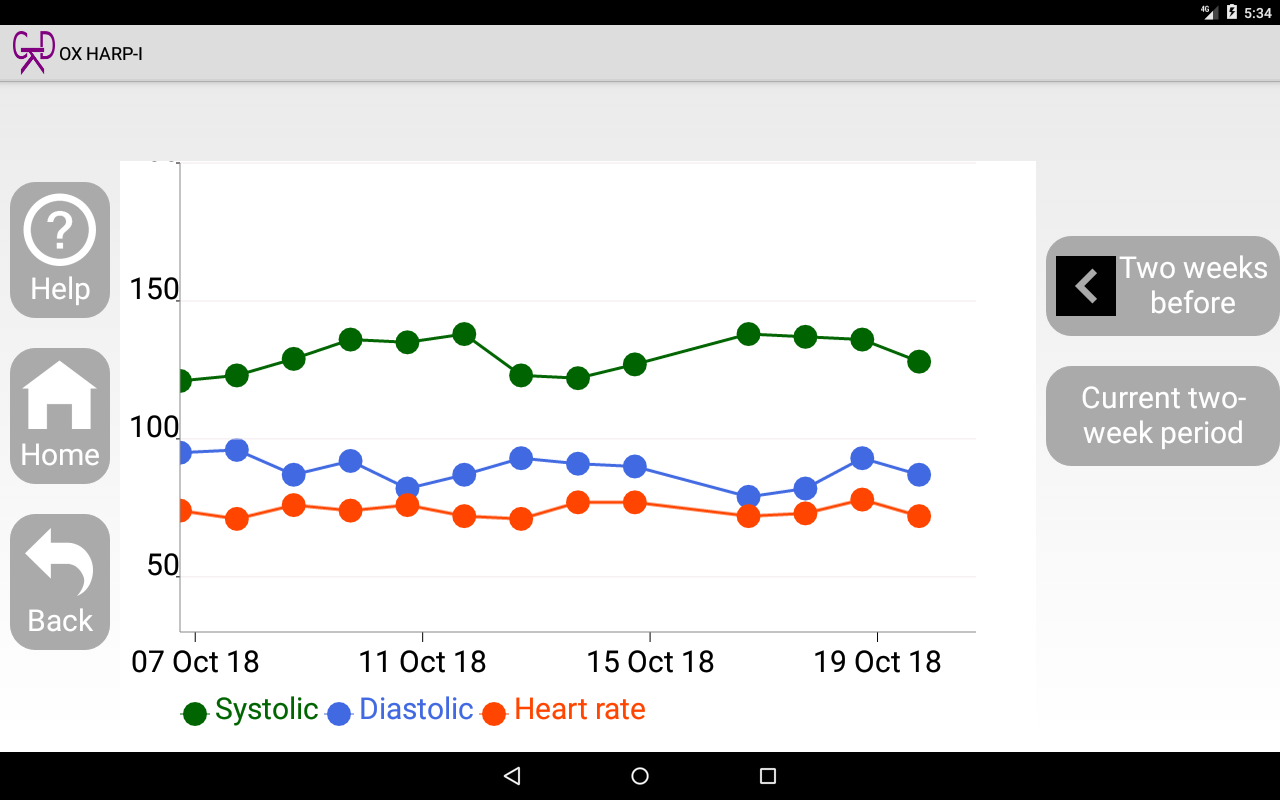

Supplement: Multimedia Appendix 4 [file cardio_v2i2e11332_app4.png]

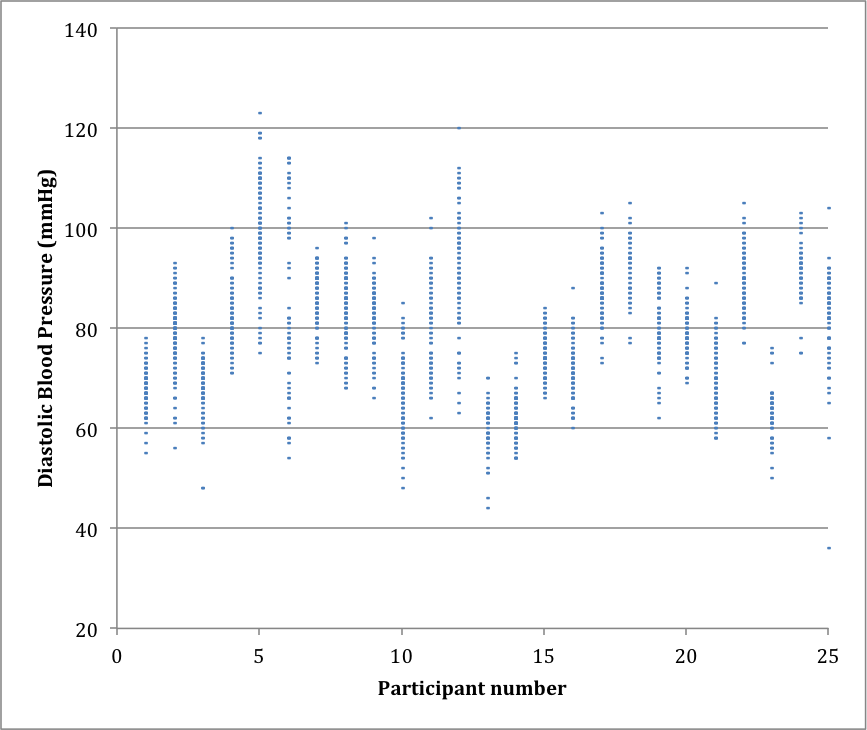

Supplement: Multimedia Appendix 8 [file cardio_v2i2e11332_app8.png]

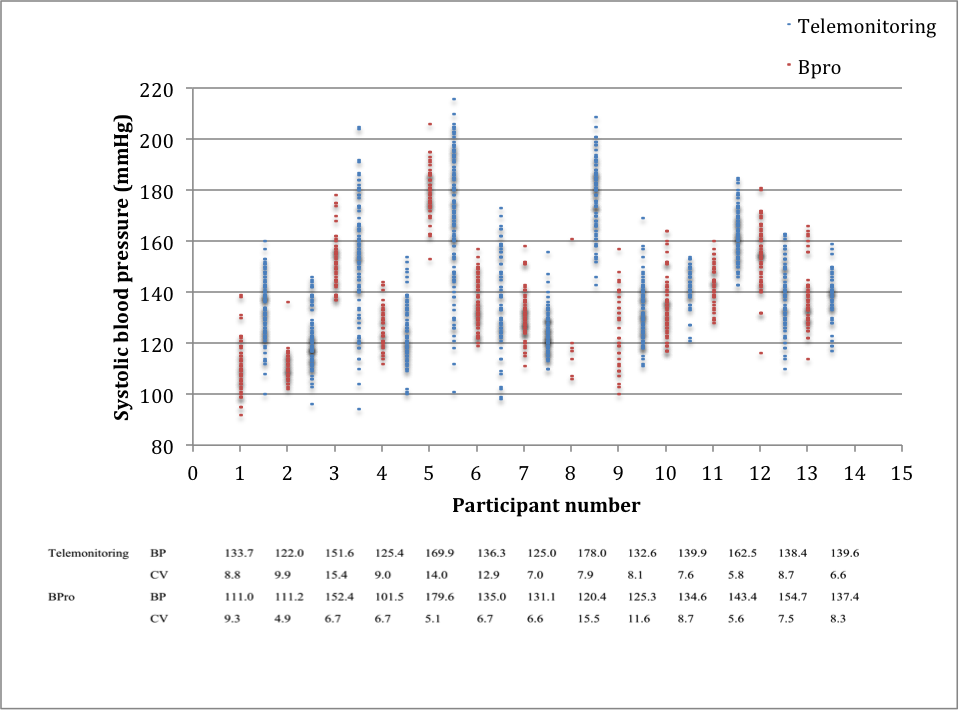

Supplement: Multimedia Appendix 9 [file cardio_v2i2e11332_app9.png]
