# Supplementary material for: Feasibility of Telemonitoring Blood Pressure in Patients With Kidney Disease (Oxford Heart and Renal Protection Study-1): Observational Study
Source: JMIR Cardio. 2018 Dec 21;2(2):e11332. doi: 10.2196/11332 (PMC6309686; doi:10.2196/11332)
Supplement: Multimedia Appendix 3 [file cardio_v2i2e11332_app3.pdf]

Questionnaire questions asked at 1 and 3 months:

1. I think that I would like to use this system frequently
2. I [do not] find the system unnecessarily complex
3. I think the system is easy to use
4. I [do not] think that I would need the support of a technical person to be able to use this system
5. I find the various functions in the system were well integrated
6. I [do not] think there is too much inconsistency in this system
7. I would imagine that most people would learn to use this system very quickly
8. I [do not] find the system very cumbersome to use
9. I [did not] need to learn a lot of things before I could get going with the system
10. I feel very confident using the system

Additional questionnaire questions asked at 3 months:

11. Using the system has improved how I manage my blood pressure
12. I would use the system to self-monitor my blood pressure long-term e.g. as part of my regular clinical care
13. I have the necessary knowledge to use the system
14. Someone can help me if I have a problem with the system
15. People who look after my health think I should use the system
16. I would recommend this system to other patients
