# Supplementary material for: Feasibility of Telemonitoring Blood Pressure in Patients With Kidney Disease (Oxford Heart and Renal Protection Study-1): Observational Study
Source: JMIR Cardio. 2018 Dec 21;2(2):e11332. doi: 10.2196/11332 (PMC6309686; doi:10.2196/11332)
Supplement: Multimedia Appendix 5 [file cardio_v2i2e11332_app5.pdf]

**Baseline characteristic**		**No. subjects (% of total)**	**Mean % BP data provided**	**% subjects providing >90% expected data**	**% subjects providing >80% expected data**
Age (years)					
	<40	2 (8%)	83.0	0.0	100.0
	>=40 <60	11 (44%)	77.5	30.0	50.0
	>=60	12 (48%)	93.3	76.9	92.3
Sex	Male	21 (84%)	83.0	57.0	76.0
	Female	4 (16%)	86.8	25.0	50.0
Smartphone owner	Yes	17 (68%)	92.2	75.0	87.5
	No	8 (32%)	83.4	38.9	12.2
