# Supplementary material for: Feasibility of Telemonitoring Blood Pressure in Patients With Kidney Disease (Oxford Heart and Renal Protection Study-1): Observational Study
Source: JMIR Cardio. 2018 Dec 21;2(2):e11332. doi: 10.2196/11332 (PMC6309686; doi:10.2196/11332)
Supplement: Multimedia Appendix 6 [file cardio_v2i2e11332_app6.pdf]

	1 month	3 months
Mean (SE) SUS score	84.9 (2.8)	84.2 (4.1)
Mean (SE) SUS score smartphone users	87.5 (2.2)	89.9 (2.7)
Mean (SE) SUS score non-smartphone users	74.7 (3.0)	72.2 (5.5)
