# Supplementary material for: Feasibility of Telemonitoring Blood Pressure in Patients With Kidney Disease (Oxford Heart and Renal Protection Study-1): Observational Study
Source: JMIR Cardio. 2018 Dec 21;2(2):e11332. doi: 10.2196/11332 (PMC6309686; doi:10.2196/11332)
Supplement: Multimedia Appendix 7 [file cardio_v2i2e11332_app7.pdf]

	**Mean (SE) Score**	**Mean (SE) score of patients with a smart phone**	**Mean (SE) score of patients without a smart phone**
Performance expectancy: Using the system has improved how I manage my blood pressure	3.84 (0.27)	4.0 (0.25)	3.5 (0.30)
Behavioural intention: I would use the system to self-monitor my blood pressure long-term e.g. as part of my regular clinical care	44.12 (0.28)	4.53 (0.21)	3.25 (0.33)
Facilitating conditions: I have the necessary knowledge to use the system	4.48 (0.21)	4.88 (0.07)	3.63 (0.30)
Facilitating conditions: Someone can help me if I have a problem with the system	3.96 (0.26)	4.18 (0.27)	3.50 (0.24)
Social influence: People who look after my health think I should use the system	3.84 (0.25)	3.94 (0.27)	3.63 (0.21)
Behavioural intention: I would recommend this system to other patients	4.4 (0.24)	4.53 (0.21)	4.13 (0.29)
